# Metallographic Evaluation of Increased Susceptibility to Intermediate Embrittlement of Engine Valve Forgings Made of NCF 3015 High Nickel and Chromium Steel

**DOI:** 10.3390/ma16196370

**Published:** 2023-09-23

**Authors:** Marzena M. Lachowicz, Maciej Zwierzchowski, Marek Hawryluk, Zbigniew Gronostajski, Marta Janik

**Affiliations:** 1 Department of Metal Forming, Welding and Metrology, Wroclaw University of Science and Technology, Lukasiewicza Street 5, 50-370 Wroclaw, Poland; maciej.zwierzchowski@pwr.edu.pl (M.Z.); marek.hawryluk@pwr.edu.pl (M.H.); zbigniew.gronostajski@pwr.edu.pl (Z.G.); marta.janik@mahle.com (M.J.); 2 MAHLE Polska, Mahle 6, 63-700 Krotoszyn, Poland

**Keywords:** iron-nickel-chromium alloy, NCF 3015, precipitation-hardenable austenitic steel, forging, carbon content, M_23_C_6_-type carbides, intermediate embrittlement

## Abstract

This paper focused on determining the increased tendency of cracking after the die forging process of high nickel and chromium steel. The increase in carbon content in austenitic nickel–chromium steel promoted the tendency of valve forgings to forging intergranular crack on the valve head. Attention was paid to issues related to the chemical composition of the material to be considered when hot forming nickel–chromium steel components. Optical and scanning electron microscopies were used to examine the microstructure and fracture features of the samples removed from a fractured valve head. The embrittlement was due to microcavity formation at grain boundaries. Creep theory at grain boundaries was used to explain crack formation. The tensile behavior was interpreted from the evolution of the microstructure during deformation and referred to intermediate brittleness to explain the effect of carbon. It was found that the increased carbon content of the nickel–chromium steel and the strong undercooling observed at the edges of the valve head are factors that promote a reduction in grain boundary cohesion and enhance intermediate temperature embrittlement. Finally, it was found that the formation of a heterogeneous structure manifested by the presence of grain boundary M_23_C_6_-type carbides in the austenitic matrix was most likely related to the occurring brittleness.

## 1. Introduction

Automotive engine valves are exploited at temperatures and stresses that promote creep phenomena and low-cycle fatigue. The combustion pressure exceeds 20 MPa, while temperatures exceed 650 °C for exhaust valves and 350 °C for inlet valves [1,2]. Exposure to increased pressure and temperature poses a major technological challenge. At the same time, increasing rigorous emission standards are becoming a major challenge in the selection of steels for automotive valves [2,3]. To provide adequate creep strength to austenitic stainless steel, simple solution strengthening is insufficient. In such situations, precipitation strengthening methods are used, mainly in the form of carbide precipitation in the form of M_23_C_6_-type carbides, (Nb, Ti)C carbides, and carbide–nitride precipitation. Therefore, NCF3015 valve steel is used for these applications [1,2,4,5]. This leads to the formation of a heterogeneous microstructure, which can lead to process problems during forging.

Austenitic steels belong to materials with low stacking fault energy [6,7]. Temperatures higher than half the material melting point ensure the occurrence of dynamic recrystallisation, which is initiated at critical stress. This has a significant effect on reducing the external forces required to shape the material, and thus facilitates the metal forming operation. The initial forging temperature of austenitic stainless steel generally does not exceed 1200 °C. The final temperature is up to 825 to 850 °C and is limited by the temperature-sensitive precipitation of M_23_C_6_ carbide. With the exception of stable and ultralow carbon stainless steels, the final forging temperature of almost all austenitic stainless steels should be controlled above the sensitive range of these temperatures and cooled rapidly within this temperature range. During hot forming, austenitic steels are subjected to several phenomena, such as work hardening, dynamic recovery (DRV), and recrystallisation (DRX). The effects of these metallurgical phenomena are often manifested in melt flow curves [8]. The influence of strain rate and temperature on microstructural changes was also observed [9,10].

**Figure 1:**
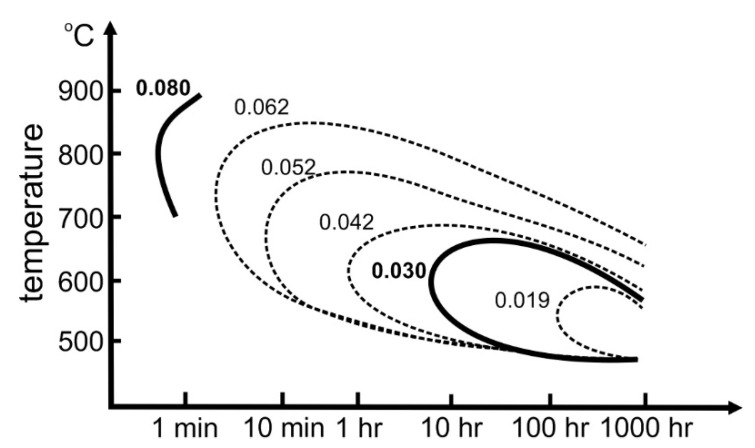
Diagram representing the temperature and time dependence of the precipitation of M_23_C_6_-type carbides on the carbon content according to [13]. The carbon content in the analyzed materials was marked in bold.

Instability in the chemical composition and inhomogeneity of steels can significantly affect the final properties of steel products. Figure 1 shows the time–temperature dependence of intergranular carbide precipitation in austenitic chromium–nickel steels as a function of carbon content. It shows a clear dependence of the temperature and time required for the precipitation of M_23_C_6_ carbides at the austenite grain boundaries (GBs) on the carbon content of the steel. Hawryluk et al. [4] studied the ranges of chromium carbide precipitation for NCF3015 steel with a carbon content of 0.04%. They found that during cooling, the first carbides could form as early as 850 °C. The higher carbon content shifts the onset of precipitation of these carbides toward higher temperatures and shorter times. The large role of plastic deformation in precipitation mechanisms is indicated as accelerating carbide precipitation processes [11,12]. The authors of the paper [6] observed the precipitation of M_23_C_6_-type in steel with 0.04%C content during deformation in the temperature range 800–850 °C. Studies also indicate that the formation and growth of precipitates are significantly dependent on the strain rate. Lower deformation rates favour the formation and growth of precipitates by promoting diffusion, which favours the formation of larger precipitates [6]. Increasing the strain rate should counteract precipitate formation. The measured strain activation energy of the austenitic AISI 321 stainless steel with a carbon content of 0.04 wt% was 453 kJ/mol in the temperature range 900–1100 °C [6]. In the range between 800 and 900 °C, a marked increase in this energy was observed, which was related to the formation of M_23_C_6_ carbides at the grain boundary (GB) of austenite.

Some studies have reported several types of intergranular cracks in the intermediate temperature range that occur during steel heating to high temperatures (intermediate temperature embrittlement). They are usually associated with cracks that occur during welding processes. When considering the mechanisms of their formation, which are closely related to the presence of tensile stresses, they can be considered not only as thermal stresses arising during welding, but also as stresses induced by plastic deformation at high temperatures. One of these is the phenomenon of *ductility dip cracking* (DDC). DDC is an intermediate temperature solid-state GB embrittlement phenomenon. For austenitic steels, the range of ductility dip associated with this phenomenon occurs between 0.5 and 0.7 of the melting points [14,15]. The phenomenon is indicated to be related to GB migration, which is favoured by the presence of stresses, and the phenomenon itself is similar to creep [14]. Materials with curly GBs are said to be less sensitive than materials with long and straight GBs [14,16]. The latter are more susceptible to sliding and migration at elevated temperatures. Also, (Nb, Ti)C precipitates limit GB mobility [17]. However, there is no consensus on the exact role of carbides and their effect on DDC sensitivity. It has been observed that the loss range of ductility corresponds to the temperature range of the release of M_23_C_6_ type carbide. Therefore, the mechanism is often associated with the precipitation of this type of carbide at the GBs. Then, in the areas where these intergranular carbides occur, nucleation of microcavities is observed [18]. Crack formation is possible as a result of creep and the merging of voids formed on the particles of the precipitates during sliding at the GBs [18]. The transition from austenite to carbide phases is accompanied by an increase in volume, which is a potential source of stress due to high strain [19]. Formation of (Nb, Ti)C carbides should reduce the amount of carbon that causes the formation of M_23_C_6_ and therefore the tendency for DCC [20]. However, on the other hand, there is a body of evidence that the presence of intergranular precipitates promotes the strengthening of the grain interior, which also translates into a weakening of the GBs [21]. It was found in [22] that the presence of precipitates at the GBs forming a carbon content of 0.024 wt% reduced the sensitivity to DDC compared to an alloy with an ultra-low carbon content. However, research was focused on additive-manufactured nickel alloy. Due to the possibility of precipitation formation at the GBs, this mechanism is also associated with the term *precipitation-induced cracking* (PIC). When discussing the role of precipitates in corrosion resistant steels, the influence of the σ phase, the presence of which can drastically affect the ductility of the steel, should also not be neglected [23,24].

Another type of cracking is called *reheat cracking* (RC) or *stress relief/relaxation cracking* (SRC), which is an intergranular failure caused by the relaxation of residual macro stresses at high temperatures [18,21]. The main mechanical condition for the appearance of RC has been found to be the presence of tensile residual stresses in the material prior to exposure to high temperatures. These stresses can be introduced during the plastic deformation of the material. Typically, the maximum tensile stresses usually do not exceed the yield strength of the tested material [25]. However, carbide precipitations can also influence this mechanism. Under long-term ageing conditions, intergranular M_23_C_6_ carbides formed in Super304H steels were found to be more prone to fracture than intergranular nitrocarbides Nb(C,N) [21].

A third mechanism that affects GB cohesion is the segregation of impurities. The presence of impurity elements, such as phosphorus, facilitates the nucleation of the creep cavity by reducing the cohesive energy of the GB [12,26]. An increased phosphorus content was found to increase the number of microcavities forming at the GBs; however, it was not the dominant mechanism responsible for intergranular damage. It was also found that the mechanisms of intergranular precipitation of M_23_C_6_-type carbide and segregation of phosphorus can influence each other. P-segregation accelerates both nucleation and growth of M_23_C_6_-type carbide participation [27].

The presence of carbides is not among the only possible causes of intergranular fracture. Another mechanism associated with intergranular damage in austenitic steels may be the result of hydrogen-induced loss of ductility [28]. The presence of hydrogen in steel has a significant effect on the strength and ductility properties of steel, which decrease significantly with an increase in the hydrogen content in the metal, contributing to the possibility of intergranular cracking. Hydrogenation occurs through hydrogen adhesion, penetration, diffusion, and local concentration in materials. Facilitated by the small diameter of the atoms, hydrogen easily diffuses into iron crystal structures even at room temperature. The resulting phenomenon is referred to as *hydrogen embrittlement* (HE). Hydrogen ingress into steel can occur during operation but also when technological processes are not carried out properly. Nevertheless, austenitic steels are considered to be among the materials that are more resistant to hydrogen ingress.

Heating steel to very high temperatures can also result in a phenomenon known as *constitutional liquation* (CL). This mechanism is closely related to the heating rate and usually occurs in the heat affected zones of massive-welded parts. It results from a combination of thermal stresses arising during rapid heating and the very low ductility of the material caused by the presence of a liquid phase in the GB. The phenomenon was proposed by Pepe and Savage, who observed its occurrence in 18-Ni steel during the welding process [29]. The mechanism is directly related to rapid heating. When the alloy is slowly heated under equilibrium conditions, the M_23_C6 carbides are completely dissolved in the matrix. However, if the heating rate of the material is high, then there is not enough time for diffusion, so that the dissolved atoms released from the partially dissolved M_23_C_6_ carbides could dissolve in the surrounding matrix. Consequently, rapid heating causes concentration of dissolved substances by dissolution in the solid state at the deposit–matrix interface. In this way, the surrounding matrix will be saturated with the elements resulting from their dissolution (chromium and carbon), causing a change in the chemical composition of the matrix adjacent to the carbides. When the local temperature and solute concentration reach eutectic precipitation–matrix equilibrium, this results in the formation of a thin film of metastable liquid at the GBs. When these liquid films merge to form a large single membrane zone, the occurrence of tensile stress can induce CL-induced cracking [30]. Residual elements such as S, P, or B may play an important role in increasing the susceptibility to CL. Carbon is considered to have an adverse effect on CL and from this point of view should be kept low. However, it is emphasised that many liquation cracks are associated with alloying elements such as niobium or titanium. The influence can be particularly significant when Ti or Nb is present. Their effect on liquation cracking susceptibility is generally unfavorable.

The aim of this research was to explain the mechanism of embrittlement that occurs with hot forging of nickel–chromium austenitic steel at intermediate temperature. The reasons for this embrittlement mechanism are not known. The formation of cracks during hot forging of steel causes economic loss during production. Identification and characterization of the origin of these defects would allow for a better understanding of the detailed embrittlement mechanism and reduced production costs.

## 2. Materials and Methods

The tests were carried out on steel used in the manufacture of a nickel–chromium austenitic steel NCF 3015 valve forging with the alloy designation UNS66315 (US). The steels tested were supplied by two suppliers, identified as D1 and D2, respectively. The chemical compositions of the steels tested, determined by the GD OES method using the GDS-500A analyzer (Leco Corporation, St. Joseph, MI, USA) are presented in Table 1. The steel belongs to the group of austenitic precipitation-strengthened steels. In the delivered state, it is characterized by the presence of chromium carbide precipitates occurring at the GBs. Microstructural studies of the tested steels are presented in the next section.

**Figure 2:**
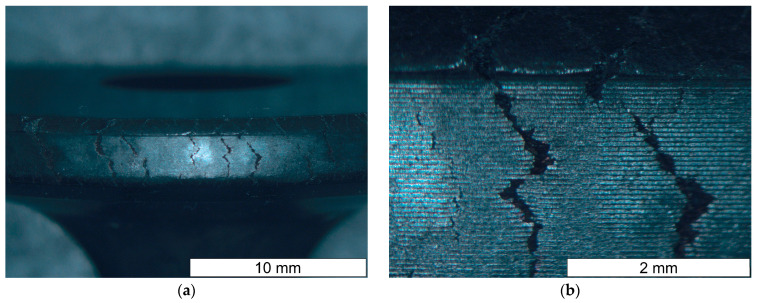
(**a**) General view of the brittle cracks formed on the valve head (margin) during forging using steel from supplier D2; (**b**) magnified section of the area from figure (**a**). Stereoscopic microscopy images.

A series of valves from supplier D2 showed a tendency to develop brittle cracks during cooling after hot forging. Figure 2 shows a general view of the example valve made of steel from supplier D2. Numerous transverse cracks were observed at the edge of the valve head. The tiny cracks were initiated in the bulged surface. Such a phenomenon was not observed in the case of valves made of steel from supplier D1.

**Figure 3:**
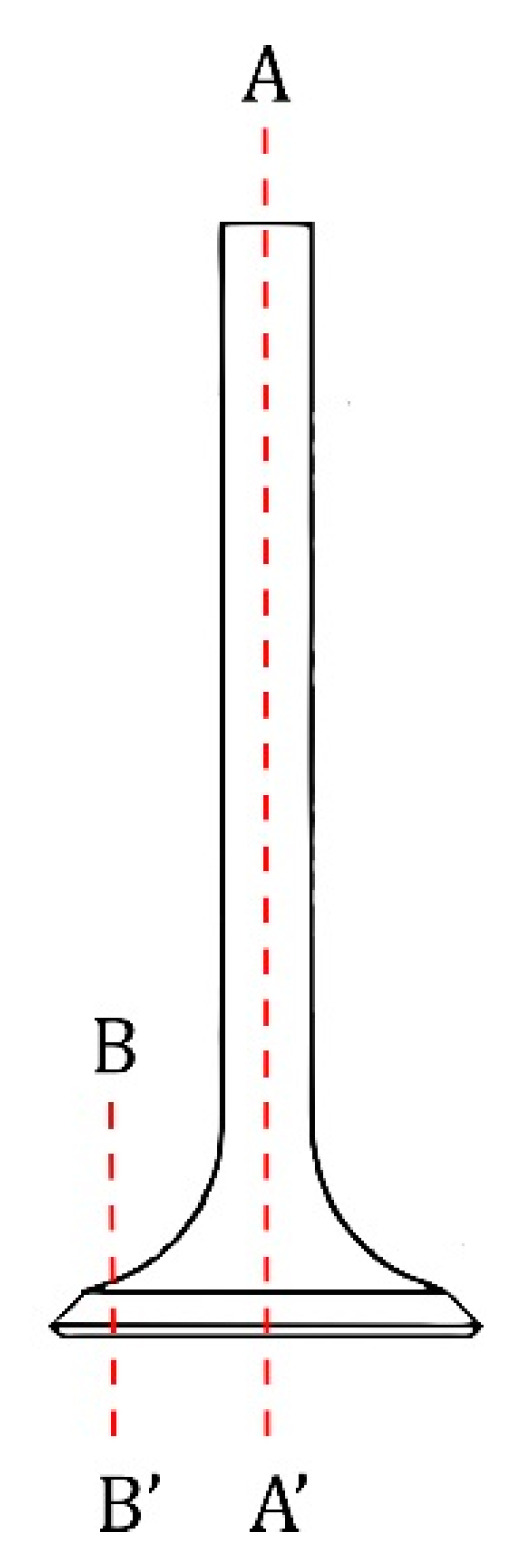
Scheme of cutting samples for microscopic examination.

The aim of the investigation was to determine the metallurgical or microstructural causes leading to the presented differences between the investigated steels occurring during valve forging. Therefore, the following research methods were applied:

1. Macro- and microstructural studies:

Observations using light microscopic imaging were carried out to compare the two materials in terms of microstructure. In order to understand the structural changes occurring in the successive stages of a properly performed forging, microscopic examinations of material D1 after induction heating were carried out, and the flow lines formed during operations I and II were determined at the macroscopic testing stage. Microstructural observations were carried out using a Leica DM6000M light microscope (Leica Microsystems, Wetzlar, Hesse, Germany). Macroscopic examinations of the fracture surfaces were carried out using a Leica M205 C (Leica Microsystems, Wetzlar, Hesse, Germany) stereoscopic microscope. The main metallographic examinations were carried out in the plane A-A′ marked in Figure 3. Macroscopic and microscopic observations were also made on a cross-section cut out of the head valve in the plane B-B′. The tests were carried out in the etched state after using the reagent of 10% oxalic acid or aqua regia (HNO_3_ + 3HCl).

A microstructural study of the cracked valve from supplier D2 was also carried out. The aim was to determine the nature of the crack formation. The light microscopic tests were extended to include detailed microscopic observations of areas from around the crack conducted using Phenom World ProX (Thermo Fisher Scientific, Waltham, MA, USA) scanning electron microscope (SEM), equipped with EDX detector. These were enriched with microanalyses of the chemical composition of the precipitates present in the material microstructure. Tests were carried out in the etched state on conventionally prepared metallographic specimens.

2. Hardness:

Hardness measurements were carried out for both materials in the as-delivered condition. The Vickers method was used to determine the overall hardness at a load of 1 kg. Once the inhomogeneity of the D2 material was established, additional hardness tests were performed for it at a load of 50 g to determine the existing differences resulting from the existing inhomogeneity. To determine the nature of the variations in hardness occurring in different areas of the valve after the forging process, hardness measurements were carried out on a cross-section of the valve from supplier D1. Hardness measurements were made with the LECO LC100 hardness tester (Leco Corporation, St. Joseph, MI, USA)

3. Static tensile test:

The research of both supplied materials involving tensile tests was carried out in accordance with ASTM A48 on a Zwick type 1478 universal testing machine with a nominal load of 100 kN (ZwickRoell, Ulm, Baden-Württemberg, Germany) equipped with the following:

- HBM rheometer (maximum range up to 165% F_nom_ of the head used)—accuracy class 0.5/1, range in accordance with the force measuring head used in accordance with PN-EN ISO 6892-1:2020-05 [31]
- macroextensometer with MT25 sensor with a measurement range of 0–150 mm and a resolution of 0.2 μm
- resistance furnace for heating (10 °C/s, in the range 0–1500 °C)
- licenced testXpert^®^ software (version 3.3.0.4258).

The initial test speed was 0.2 mm/min. Once the force sufficient to determine Young’s modulus had been reached, the test execution speed was increased to V = 2 mm/min. Total elongation was determined from the extensometer for a 50 mm test base. Standardised tensile test specimens were made from input material supplied by both suppliers, D1 and D2. In the case of supplier D2, in addition to testing the material in the condition as-delivered, tests were also carried out on samples after heat treatment consisting of annealing at 200 °C for 24 h. The objective of the heat treatment was to assess the possible dehydrogenation of the samples from supplier D2. The tests were carried out on three samples for each of the tested materials. 

## 3. Results

### 3.1. Characterisation of the Material as Delivered

#### 3.1.1. Microstructural Examination

**Figure 4:**
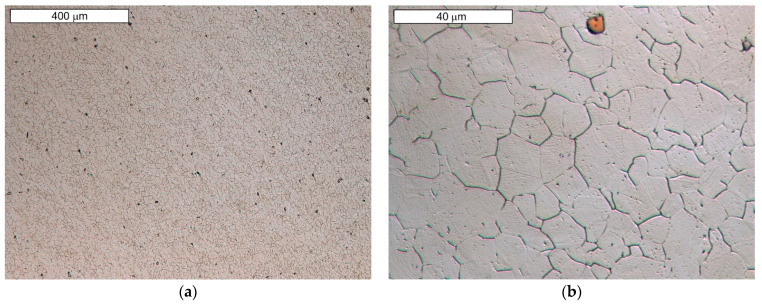
(**a**) Microstructure of steel from supplier D1; (**b**) magnified section of the area from (**a**). Light microscopy, etched with 10% oxalic acid.

**Figure 5:**
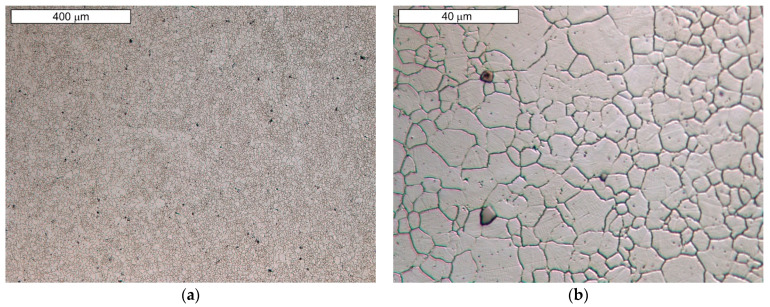
(**a**) Microstructure of steel from supplier D2; (**b**) magnified section of the area from (**a**). Light microscopy, etched with 10% oxalic acid.

Microscopic observations carried out for both materials showed differences in their austenite grain size. The material from supplier D1 was characterised by a slightly larger but more homogeneous grain size (Figure 4). The equivalent index of grain size corresponded to G = 9.5 according to the EN ISO 643 standard [32]. Material from D2 was characterised by significant heterogeneity in grain size (Figure 5). In the case of supplier D2, 70% of the grains corresponded to the G10 index, while the remaining 30% corresponded to the G8.5 index in accordance with the EN ISO 643 standard [32]. However, it should be noted that very fine grain size was observed for both materials. The fineness of the grain size can effectively increase the strength of the steel, which is also commonly known as Hall–Petch strengthening. However, GBs also act as preferential sites for crack nucleation and GB migration, especially at elevated temperatures. Larger initial grain sizes favour the delayed onset of dynamic recrystallisation (DRX) [33], which affects the flow stress of the material. The occurrence of DRX in a material with a small grain size is more likely due to an increase in the density of nucleation sites for new grains [7]. Fine M_23_C_6_ particles delay or hinder the recrystallisation process, while large M_23_C_6_ particles stimulate recrystallisation [34]. Large particles of alloy carbides (Ti, Nb, Mo, Zr)C as well as titanium nitrides were also observed in the microstructure. The authors presented research on phase identification in the work [4]. The distribution of elements carried out using the EDS method for both types of carbides is also presented later in the work. As mentioned earlier, precipitates of (Nb, Ti)C inhibit the sliding of GBs [17]. For this reason, some authors indicate that elements that form strong MC-type carbides, such as Nb and Ti, reduce the sensitivity to DDC. It has been indicated that these elements, by increasing the amount of primary carbides, lead to the formation of winding GBs that hinder DDC propagation. (Nb, Ti)C precipitated at GBs inhibits cavity coalescence at high temperature [17]. Consequently, it has been suggested that cracking can be mitigated by increasing the niobium and titanium content. It has also been indicated that DDC can be limited by reducing the mismatch between matrix and carbide precipitates by lowering Cr and Fe concentrations, as well as by minimizing global stresses (caused by welding or deformation) [20]. 

On analysing the chemical composition of the tested materials (Table 1), it can be concluded that the niobium and titanium contents are similar, which is why the increased carbon content should not contribute to the increased niobium carbides and titanium carbides present in the microstructure of the tested steel. No significant qualitative differences in these precipitates were observed between the tested materials. Carbon translates into solution strengthening, as confirmed by the strength tests presented later in this article. Carbon strengthens the steel interstitially, which clearly translates into a strengthening effect.

When supersaturated austenite is cooled, the excess carbon released is bound into carbides, and the tendency of the steel to precipitate carbides during cooling is dependent on the carbon concentration in the steel. At higher carbon content, carbide release requires a shorter time and is also initiated at higher temperatures (Figure 1). Diffusion is easier at GBs, with the result that precipitates appear faster at GBs. Pommier et al. [18] found that the formation of M_23_C_6_-type carbides at the GBs of 316L steel with a carbon content of 0.028% promoted a reduction in GB strength compared to steel containing 0.011% C. These carbides formed at the misoriented GBs from 25 to 55°.

#### 3.1.2. Hardness of the Material

The observed differences resulted in a higher material hardness of D2, amounting to 399 ± 8 HV1. The average hardness of D1 material was 347 ± 5 HV1. The observed heterogeneity in grain size in the material from D2 also resulted in a variation in microhardness in different areas of the material. It averaged around 400 HV0.05 in areas with larger grain size, while it ranged from 468 to as much as 528 HV0.05 in areas with very fine grains, whose overall larger surface area resulted in the accumulation of chromium carbides present in these areas. A similar structural effect associated with grain inhomogeneity was observed by the authors of work [35] in 5Cr21Mn9Ni4N grade steel.

#### 3.1.3. Temperature-Dependent Tensile Test

**Figure 6:**
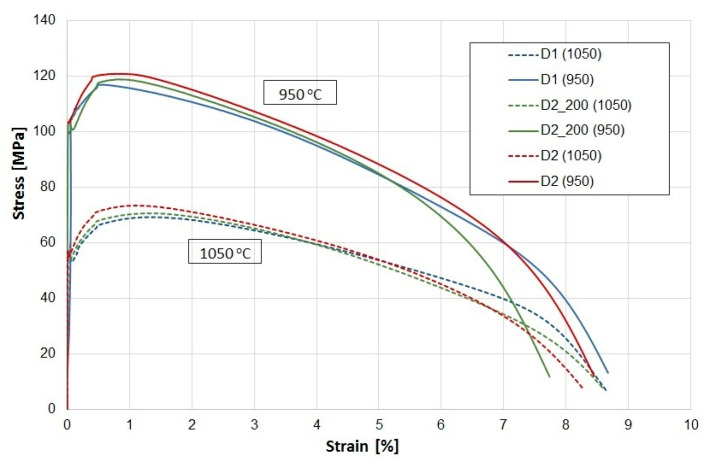
Stress–strain diagram obtained for the tested materials at different temperatures.

The stress–strain curves obtained for both materials at high temperatures are shown in Figure 6. The research was carried out on three samples, and the results obtained were consistent. The curves are shown for one of the example samples. The ductile properties of the steels are significantly affected by the presence of hydrogen in the steel, which strongly decreases with increasing hydrogen content in the metal, contributing to the possibility of intergranular cracking. To exclude this mechanism, both materials were dehydrogenized by heat treatment. For this purpose, a batch of material was subjected to 24 h of annealing at 200 °C. However, no clear differences were observed in the forging process carried out on the material without dehydrogenation and after hydrogenation. There were also no differences between the annealed samples (D2_200) and the delivered samples (D2) in the static tensile test. If the material exhibited hydration characteristics, this would have resulted in a lower elongation. This excludes this mechanism as responsible for valve fracture.

The tensile curves obtained allow the conclusion that a higher carbon content in the steel from D2 translates into higher strength parameters. In addition to solution strengthening, the strength may be influenced by a lot of strengthening processes, such as strain or grain boundary strengthening, and strengthening associated with the presence of precipitates. In the analyzed case, the influence of strain hardening was excluded because the material is characterized by a microstructure typical of the annealed state. As mentioned earlier, the chemical composition analysis shows similar content related to the elements forming MC-type carbides well as titanium nitrides. Qualitatively, no significant differences were observed for both materials in this respect, which was also confirmed by microscopic observations. For this reason, the impact of these precipitates on strength would be similar in both materials, which allows their impact to be omitted from the considerations. Differences occur in the grain size, which, according to the Hall–Petch relationship, could translate into strength. However, it should be noted that the material with lower strength should have a larger grain size. In the analysed case, the D2 alloy had non-uniform grain-size areas with very fine grains and areas with much larger grains than the material from supplier D1 being observed. It cannot be completely ruled out that these differences influenced the strength. However, it seems that the main mechanism causing the increase in strength that should be considered is solution strengthening. The higher the temperature, the more evident these differences in strength were. Furthermore, slightly higher elongations can also be observed for the D1 provider compared to D2, which allows us to conclude that the deformability of the D2 material is lower (even after dehydrogenation) than for D1 without additional treatments.

### 3.2. Characterisation of the Valve Forging Process

**Figure 7:**
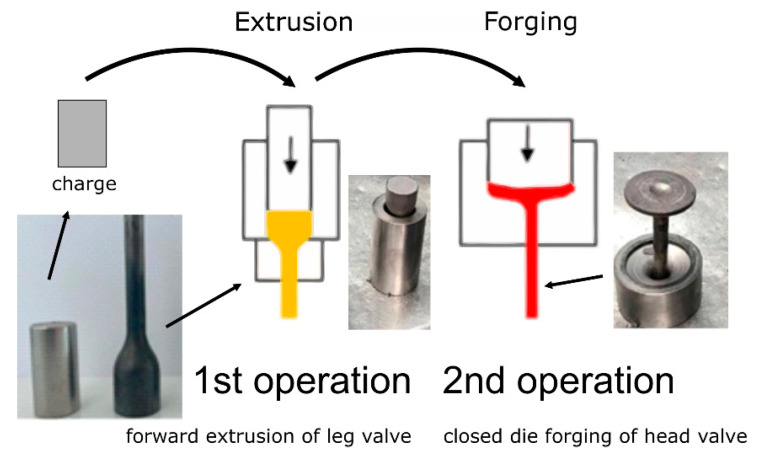
Diagram presenting two-stage fine forging process of a valve forging.

To understand the macro- and microstructural changes occurring during the forging process, microstructure analyses were carried out at various stages of the process. The valve is produced by the fine forging process in two closed die operations (Figure 7). A forging of this type is used as a key component for engines in trucks, and therefore high-performance properties are required, which are mainly related to ensuring the correct flow of the forging material during shaping, the absence of surface and internal flaws, high quality, and dimensional and shape accuracy. The forging is made in two stages: in the first step, a so-called ‘pear’ is coextruded from a roller heated to 1050 °C. The transport of the heated charge material inside the press takes place to the first cavity, where the roller is dropped into the preliminary die (1st operation), in which the valve stem is shaped using the hot extrusion process. This is followed by the second stage (2nd operation), where the valve head is forged in seamless dies. For this purpose, the forging is transported to the die located in the second cavity, where the forging process takes place. The element obtains a shape similar to the finished product.

To obtain the required temperature of the material before forging, induction heating was performed at 1050–1080 °C. The charge materials were heated in an induction heater, where the appropriate length of the inductor, power and press cycle time guarantee optimal parameters. The temperature was controlled for each editorial using a pyrometer. Forging conditions were constant for both tested samples. The method of cooling the samples after induction heating was the same in terms of cooling rate as in the case of valves, i.e., cooling in water. 

**Figure 8:**
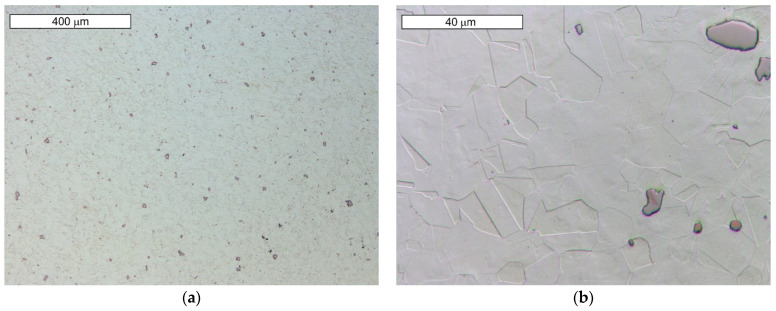
(**a**) Microstructure of the material after induction heating; (**b**) magnified section of the area from (**a**). Light microscopy, etched with 10% oxalic acid.

To assess the regularity of the microstructure of the material at this stage of the process, a microscopic examination of the material was carried out after the preforms were heated. The microstructure of the preforms after induction heating showed the presence of polygonal austenite grains with a grain size larger than the supplied material. The equivalent index of grain size corresponded to G = 8.5 according to the EN ISO 643 standard [32]. M_23_C_6_-type carbides were fully dissolved due to heating (Figure 8). Grain boundaries were slightly etched, indicating the absence of secondary phases at the grain boundaries. So, the carbides disappeared, and the grain size increased compared to the as-delivered state. For this reason, the microstructure present as delivered did not contribute to cracking of the valves during their cooling after forging. Precipitates of primary carbides and carbide–nitrides of the alloying elements were visible within the grains. A detailed metallographic analysis of the microstructure can be found in our work [4].

**Figure 9:**
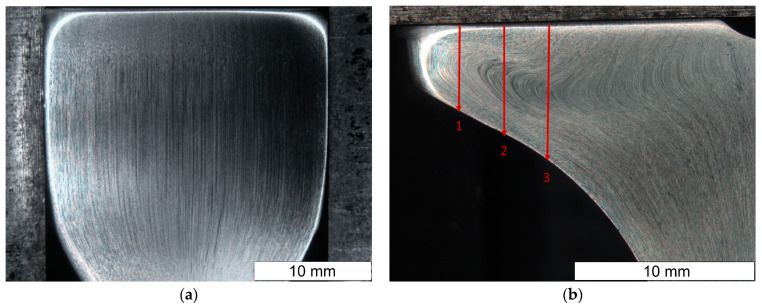
Macroscopic structure of the valve after the first (**a**) and second (**b**) stages of the process. Plane A-A′. Stereoscopic microscopy images.

To determine the course of the flow lines formed during the hot forging process of automotive valves, metallographic examinations were made on the cross section of the forging after the first and second operations. The tests were carried out on a normal valve forged from material supplied by supplier D1. The general appearance is shown in Figure 9. The arrangement of flow lines observed on the macro-sections was correct and in line with the shape of the component, which will positively influence the achieved strength of the valve. No structural abnormalities leading to packed flow lines and stress concentrations were observed. After the first operation, the flow lines were compressed together at the cross-sectional change (Figure 9a). Cracks appeared after the second operation; therefore, further tests were carried out on the final product. The flow lines were clearly narrowed in the valve stem area, due to the significant reduction in cross-sectional area that occurred in this area of the valve. They were created at the stage of the first operation. During the second operation, there was a change in the flow direction of the material in the area of the valve head (Figure 9b).

**Figure 10:**
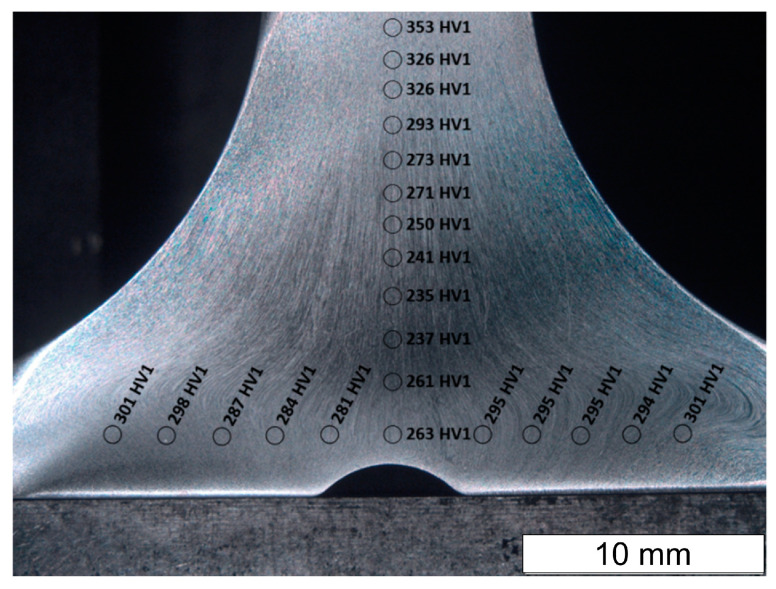
Hardness distribution obtained in selected areas of the forging after operation II. Plane A-A′. Stereoscopic microscopy images, etching with 10% oxalic acid.

**Figure 11:**
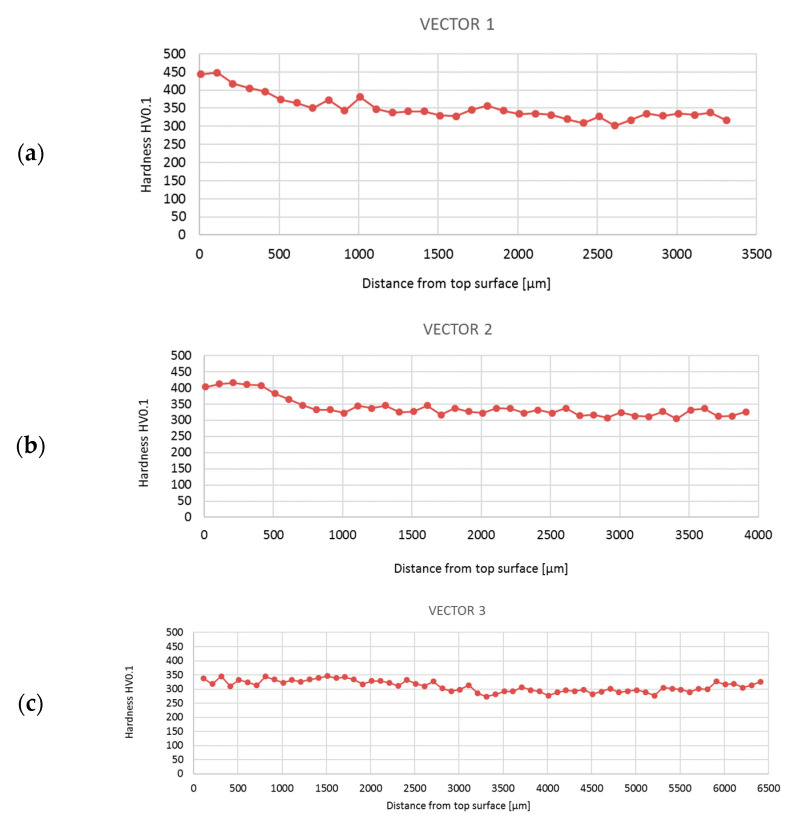
Hardness distribution obtained in selected places of the forging after the second operation for line 1 (**a**), line 2 (**b**), and line 3 (**c**) shown in Figure 9.

Hardness measurements taken in selected areas of the forging after the second operation showed variation in hardness (Figure 10). The lowest hardness was recorded in the central part of the valve head, while the highest hardness was recorded in the valve stem. High hardness values were also observed at the edge of the valve head. Due to the variation in hardness present, hardness distributions in the areas of forming cracks were also measured (Figure 11). The tests were carried out along the lines shown in Figure 9b. The highest hardness of approx. 450 HV0.1 was observed in the initial range of vectors 1 and 2. This sudden packing together of the flow lines causes an increase in hardness. The result was an increase in stress in this area. This is particularly important when the material shows brittleness. In ductile materials, local yielding will allow for stresses to be redistributed and will reduce the stress concentrations. In the further section of vectors 1 and 2, the hardness value was stabilized at the level of 350–300 HV0.1. Along the entire length of vector 3, the hardness proved constant with values oscillating around 300 HV0.1. These observations are compliant with the distribution shown in Figure 10.

**Figure 12:**
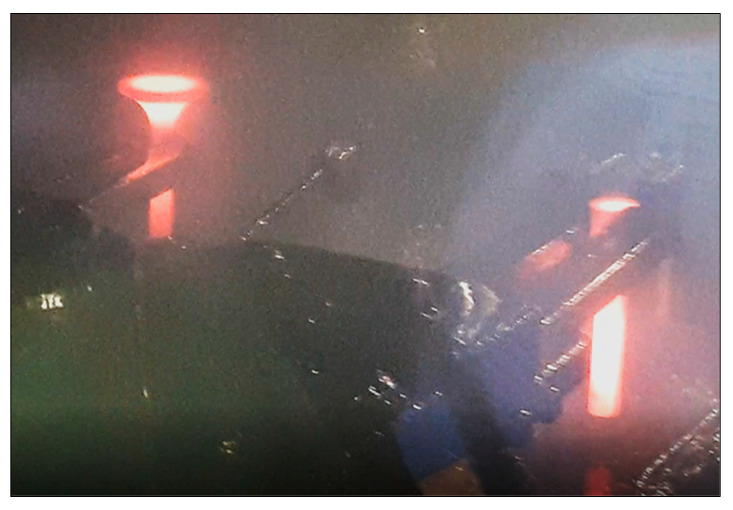
Change of valve head color during the II process indicating accelerated cooling-down in the valve.

**Figure 13:**
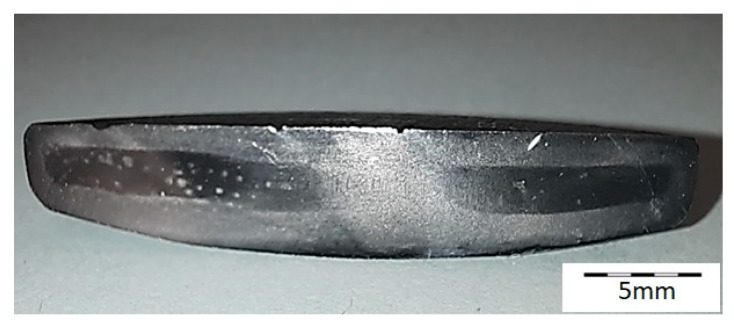
Visible two zones in the cross section made on the valve head. Plane B-B′. Stereoscopic microscopy images, etching with aqua regia (HNO_3_ + 3HCl).

**Figure 14:**
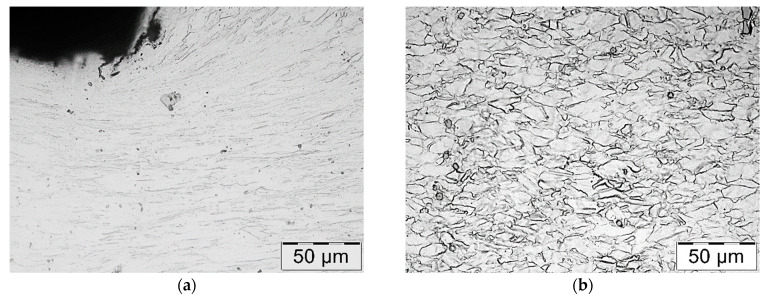
Microstructure of the material in the outer (**a**) and inner (**b**) zones shown in Figure 13. Light microscopy images, etching with aqua regia (HNO_3_ + 3HCl).

Figure 12 shows a photograph taken during the course of the process. It can be observed that the area of the valve head’s edge is significantly more overcooled than that of the other part of the valve. This is indicated by the darkening of the steel observed in this area. It should be noted that this temperature drop occurs in the area of high stress concentration, as indicated by the higher material hardness. Taking this into account, a cross-sectional examination of the valve head was carried out, which revealed the presence of a near-surface layer. Its thickness was approximately 1 mm (Figure 13). Therefore, lowering the temperature in this area has an impact on the microstructure present in it. Microscopic examinations carried out in these areas showed that the microstructure in the near-surface layer was marked by austenite grains strongly deformed and elongated along the rolling direction, whereas the core of the valve head was characterised by polygonal grains of fully recrystallised austenite. This was also confirmed by the microscopic observations shown in Figure 14. The grain size of recrystallized austenite is a function of temperature and deformation rate. The recrystallization temperature depends very clearly on the degree of previous deformation. The recrystallization process at a higher temperature range occurs in a shorter time. Ensuring evenly distributed plastic deformation and the same temperature throughout the element, allows the homogeneity of the microstructure after recrystallization to be ensured. However, in the case of forgings with a complex form and different thickness, the plastic deformation is not evenly distributed, so that the size of the recrystallized austenite grains in different areas is usually diversified. Also, the rapid deformation during the second operation and the breaks needed to transfer the produced element between operations do not create favorable conditions for the full course of recrystallization in the near-surface area.

### 3.3. Testing a Fractured Valve 

**Figure 15:**
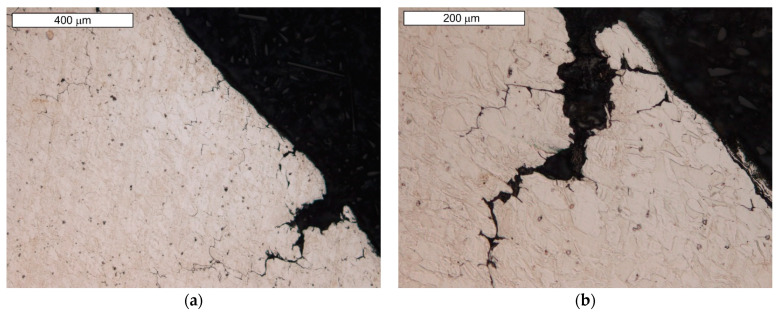
(**a**) Visible macrocracks developing along austenite grain boundaries; (**b**) magnified section of the area from (**a**). Light microscopy images, etching with 10% oxalic acid.

**Figure 16:**
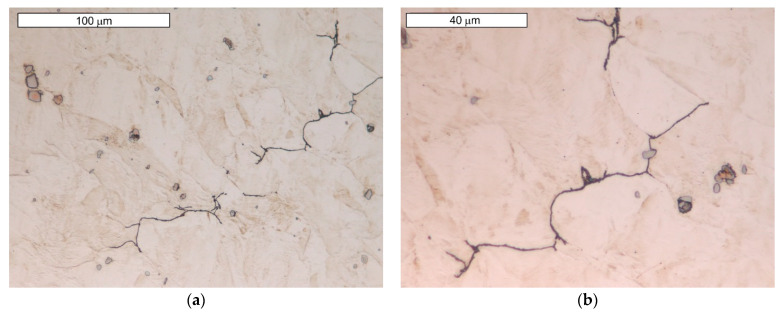
(**a**) GB decohesion leading to the formation of post-GB cracks; (**b**) magnified section of the area from figure (**a**). Light microscopy images, etching with 10% oxalic acid.

**Figure 17:**
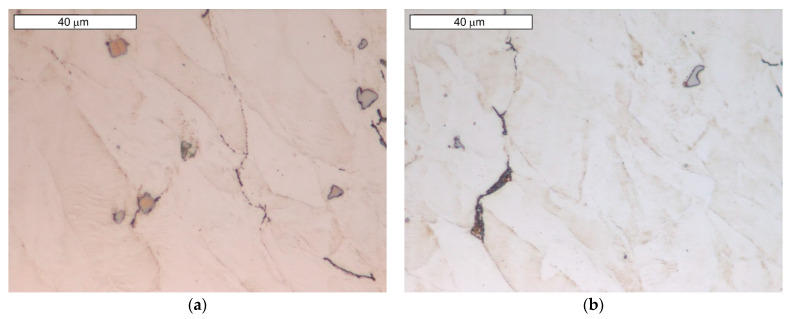
Decohesion of GBs. Visible bypassing of carbide precipitates (**a**) and local formation of an extended fracture surface (**b**). Light microscopy images, etching with 10% oxalic acid.

Microscopic examinations performed on the face of the valve head showed that the cracks observed in the macroscopic stage develop and run along the grain boundaries (GBs) of austenite (Figure 15). The neighboring micro-cracks merged and coalesced into large cracks. A decohesion of the GBs was observed, leading to the formation of clenched cracks occurring at a slight distance from the surface (Figure 16 and Figure 17). 

**Figure 18:**
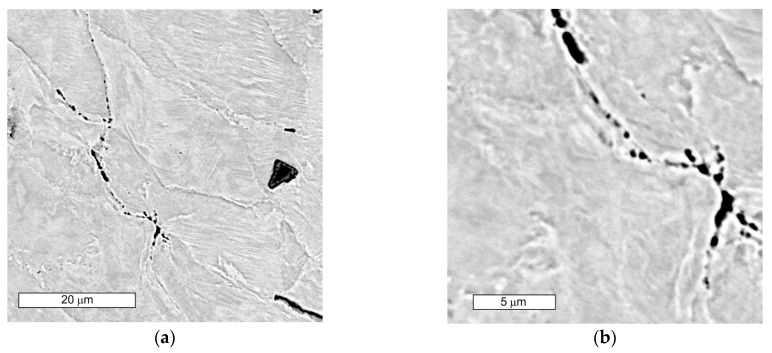
(**a**) Visible nucleation of microcavities at the boundary of austenite grains; (**b**) magnified section of the area from (**a**); SEM images.

**Figure 19:**
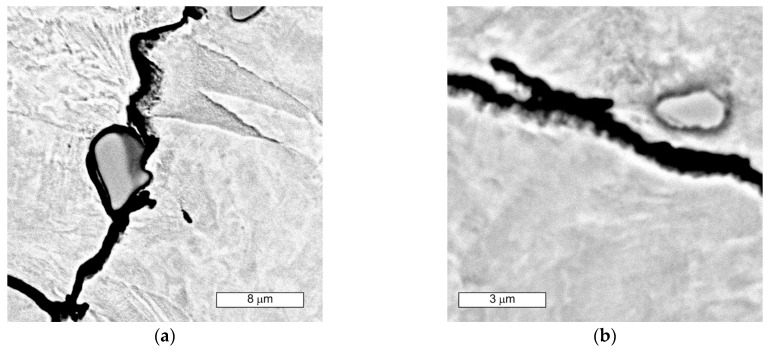
Visible “jagged” GB with a surface shaped by microcavities formed during crack development in area 1 (**a**) and 2 (**b**), SEM images.

**Figure 20:**
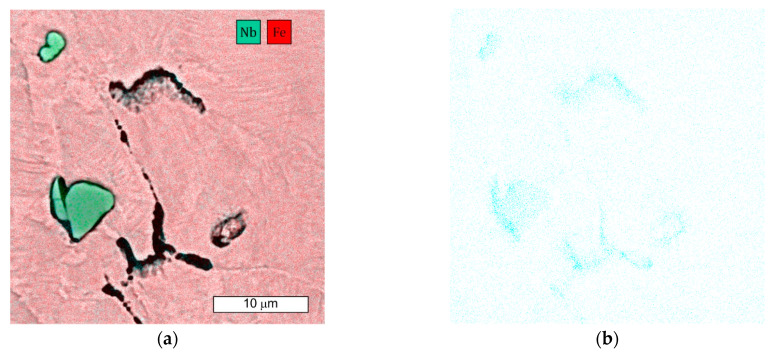
(**a**) SEM microscopic image of a composite with Fe and Nb distribution. Visible ‘frayed’ GB at the site of intergranular crack progression and intragranular carbide MC precipitation; (**b**) carbon distribution in the examined area.

SEM observations of microcavities show that they result from microvoid coalescence formed on the GB (Figure 18). The microvoids nucleate and then grow assisted by strain. Eventually, they merge, a crack is formed, and the material fractures. The formation of voids should be associated with the accumulation of dislocations near the migrating boundaries of thermally activated grains [14]. As a result of stress relaxation, GBs with extensive accumulations of dislocations serve as preferential sites for the initiation of microcavities and associated fractures [36]. GBs cannot tolerate stress redistribution that leads to boundary decohesion. Microscopic observations show that in the early stages of crack development, the formation of characteristic “bridges” is observed, which maintain GB cohesion. Disruption of this bridging leads to GB decohesion and eventual crack development. The formation of multiple intergranular cavities results in crack propagation. In order to bridge the crack with the microcracks that occur before the main crack, much lower stresses are needed compared to normal ductile tearing. Such a crack development results in GBs characterised by a jagged contour at the site. The surfaces of these cracks do not show plastic deformation (Figure 19 and Figure 20a). The fracture surface in the microscopic image shows many irregularities caused by the formation of microcavities. Such intergranular cavities and microvoids on the fracture surface were also observed by other authors [14,37]. Damage development depends on stress relaxation and GB structure, which are directly related to the chemical composition of austenitic stainless steel. Such preferential cavity generation has also been observed in austenitic steels and nickel alloys by other authors [14,17,18]. Pommier found that microcavity nucleation occurs in areas of high residual stresses due to the presence of intergranular M_23_C_6_ carbides [18]. The appearance of carbides results in a discontinuous stress distribution at the intergranular carbide–matrix interface [21]. Hence, micro-cracks initiate at this weakest interface under tensile stress. Some authors [21] state that the presence of intergranular particles can induce intergranular strengthening and weakening of the boundary zones. This results in a difference in strength between GBs and grain interiors. When the intragrain areas are strengthened by the particles within them, deformation is confined to a weak, narrow GB area. This forces the relaxation of residual stresses by creep deformation on the GB. This is followed by a concentration of deformation at the GB [21]. Due to the weaker GBs, intergranular cracking occurs.

The plastically deformed austenite provides more places suitable for carbide nucleation, occurring not only at GBs, but also at intersecting slip bands. It also provides an increased rate of diffusion, which accelerates the precipitation of the carbide phases. As M_23_C_6_ carbides are preferential locations for microvoid nucleation, the higher carbon content of steel favours their formation [18]. The occurrence of M_23_C_6_-type precipitates in GB was not observed in the SEM microscopic image. However, this does not mean that their occurrence should be ruled out. The more so, as increased carbon content was observed in these areas, which indicates its diffusion (Figure 20b). 

The single and irregular particle visible at the GBs in grey contrast is MC carbide (Figure 19). Niobium is the main component of these carbides (Figure 20a). The crack that spreads across the GBs bypasses the precipitations without compromising their cohesion. However, it should be remembered that by strengthening the grain interior, they contribute to the weakening of GBs [21].

**Figure 21:**
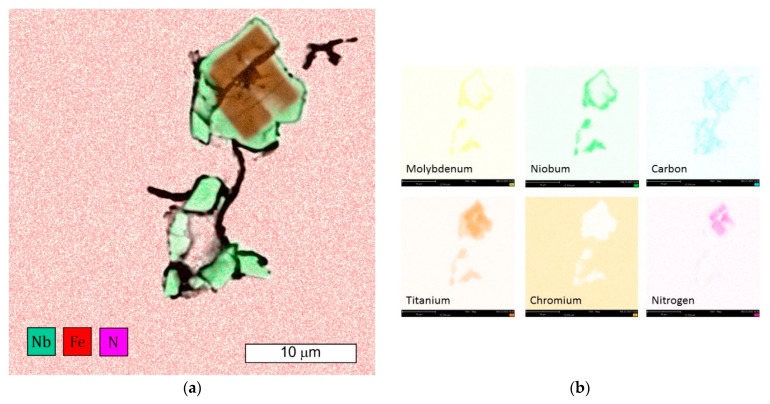
(**a**) Visible cracks occurring around the large particles present in the matrix, as well as cutting through the particle; (**b**) elemental distribution obtained from precipitation. EDX/SEM images.

Microscopic observations indicate that there were also some intracrystalline microcracks nucleating in large-size precipitates, suggesting their damaging role in fracture. Li et al. [38] indicated that a large number of MC-type particles can cause brittle steel fracture. The propagation of the crack follows the interfacial boundaries [39]. The large difference in elastic modulus that occurs between carbide and matrix is seen to be the main cause of cracking [19,40]. In this case, cracks are observed at the MC-particle–matrix interface, and then the crack propagates further. Figure 21 shows an example of the complex precipitation observed in the microstructure. The inner part is TiN, while the outer carbide is composed of Mo, Nb, and Ti, elements which form MC-type carbides. Chromium is not present in this particle. The formation of such complex particles results from the fact that TiN particles are high-temperature precipitates and fully precipitated before MC-type particles are formed [41]. For these particles present in the matrix, an observable tendency occurs to inhibit cracking. Cracks also occurred in the carbide and extended further into the matrix. Similar observations were made by the authors of the paper [19]. Simultaneously, the progressive stress concentration in their surroundings leads to the formation of cracks in the carbides themselves. This is due to the high stresses induced at the carbide–matrix interfacial boundaries, which result from the high hardness and brittleness of these carbides.

## 4. Discussion

Based on the results of the experiments, we suggest a cracking mechanism of the engine valves at the forging temperature. The formation of intergranular cracks during plastic deformation at high temperatures is associated with a significant reduction in the ductility on the GB (grain boundary). SEM microscopic studies showed that progressive merging of voids formed on the GB was a characteristic feature of cracks forming. The phenomenon of cavity formation is considered to follow a creep-like course and involves dislocation sliding. The presence of obstacles in the slip-plane hinders the slip process, which is directly responsible for the formation and growth of microcavities. Dislocation pile-up and diffusional relaxation occur along the GB. This mechanism has traditionally been attributed to stress concentrations generated at the obstacles, which may be due to the following: precipitation of intergranular carbide phases, shear stress relaxation by local grain deformation, or GB migration. The strain concentration occurs at the GBs adjacent to the carbide precipitates, which are much weaker than the grain interior. The plastic deformation that occurs is a factor that further supports these processes. Any deformation occurs via GB sliding. Increasing the carbon content of the austenitic steel raises the tendency to planar slip [42]. It was found that in low carbon steels, phosphorus segregation may be a contributing factor, which additionally reduces GB strength [43].

The research indicates that the material that underwent fracture during forging exhibited an increased carbon content, which was reflected in an increased solid solution strengthening of the steel tested in the delivery state. Analysing the times required for the initiation of carbide precipitation, as well as the high temperatures, allow the conclusion that the carbon content present in the material also increases the probability of nucleation of M_23_C_6_ carbide precipitation, which reduces the cohesion energy of the GBs. Although no precipitates were observed in the GB areas, the possibility of nucleation of finely dispersed carbide precipitates, which are coherent with the matrix and which are invisible in the light microscopy and SEM images, cannot be excluded. The valve head edge should be expected to be a favorable area for the initiation of precipitation processes occurring during forging. This is because of the high overcooling occurring there, as well as the significant plastic deformation, which promotes the migration of GBs over long distances and the strain of the material in this area. At the same time, as the study shows, the near-surface area of the valve head is characterised by higher hardness and microstructure of partially recrystallized austenite compared to the centre of the valve head. This is an additional factor that promotes relaxation of residual macro-stresses in this area during deformation at high temperatures. The mechanism known as DDC is strongly related to GB migration, and the edge of the head is the area accompanied by the highest displacements during forging. However, it is also important to keep in mind that an increase in carbon content enhances the strength of austenite, which may further promote the loss of GB cohesion. It should also be taken into account that the D2 material was characterised by microstructural heterogeneity, which meant that locally the carbon content could be higher than the average content in the steel. Increased carbon content will also favor easier precipitation of chromium carbides in service at high temperature, which will promote accelerated deterioration of the valve [35]. The very initiation of diffusion processes associated with the preparation for carbide formation may be sufficient to reduce GB cohesion.

To summarise the research conducted, the following can be concluded:

- If the presence of internal stresses present in the material prior to forging was the cause of crack formation at the heating stage (RC mechanism), then a higher randomness of the locations where cracks occur would be expected. Instead, they occur only in the most stressed area, i.e., at the edge of the valve head, which allows this mechanism to be ruled out.
- No increase in phosphorus content was observed in the chemical composition of the material of the tested materials, which also allows the exclusion of segregation of impurities as the main cause of cracking. However, it should be noted that phosphorus may intensify the negative effect of carbon in this respect.
- The presence of intracrystalline particles (such as carbide nitrides and carbides of titanium, niobium, and molybdenum) can induce a strengthening of the grain interior, which may contribute to a reduction in the strength of the boundary areas with respect to the grain interiors. This is because the presence of these particles increases the resistance to deformation and reduces the ductility. Also, strong grain interiors induced by increased carbon content can cause deformation concentrated at the GBs.

The microscopic images did not reveal the presence of low-melting components of the structure. Furthermore, the limited heating temperature of the steel before forging excludes the possibility of CL as the mechanism responsible for fracture. The scope of the occurrence of this phenomenon is definitely due to higher temperatures.

It is difficult to unequivocally say which of these mechanisms had a decisive influence on valve cracking during forging. Most likely, all of these factors contributed to the cracking simultaneously. Hence, the austenite deformation mechanism requires additional research to determine the role of the chemical composition and microstructural inhomogeneity on hot forging. In particular, this concerns the role of carbides, leading to the formation of the heterogeneous microstructure of the alloy. However, regardless of the final mechanism, it can be seen that the direct cause of cracking was the carbon content being in the upper range provided for this steel.

## 5. Conclusions

The mechanism of embrittlement of a nickel–chromium steel containing a large proportion of alloying elements, which results in heterogeneity of the forming microstructure, was studied. The characteristic embrittlement behavior was observed in the forging temperature range. Crack formation is related to changes in ductility and the nature of the stress state in the ongoing structural transformation process. The increased carbon content present in the steel material supplied by the D2 supplier should be considered the direct cause of crack formation in the material NCF2015 during valve forging. Here we report that, by careful control of the carbon content in the alloy, the tendency to crack the valve forgings can be reduced. First, the increased carbon content favours the precipitation processes occurring in the steel, creating microstructural heterogeneity. When the final forging temperature in the head area is in the temperature range where carbide nucleation is possible, just before or during deformation, this causes a susceptibility to valve cracking. This was also confirmed by in-service tests currently being carried out. Future experimental studies will focus on further structural studies. In particular, it is planned to carry out research using transmission electron microscopy (TEM) methods.

## Figures and Tables

**Table 1:** Chemical composition of the steels tested.

Element	C	Si	Mn	P	S	Cr	Ni
Supplier D1	0.025	0.24	0.34	0.006	0.002	14.53	31.6
Supplier D2	0.078	0.26	0.13	0.012	0.001	13.92	31.2
Requirements	Max. 0.08	Max. 0.5	Max. 0.5	Max. 0.015	Max. 0.001	13.5 ÷ 17.00	30.0 ÷ 33.5
**Element**	**Mo**	**Al**	**Ti**	**Nb**	**B**	**N**	**Fe**
Supplier D1	0.60	1.92	2.51	0.58	0.004	0.004	balance
Supplier D2	0.66	1.87	2.50	0.61	0.002	0.006	balance
Requirements	0.4 ÷ 1.0	1.6 ÷ 2.2	2.3 ÷ 2.9	0.4 ÷ 0.9	0.001 ÷ 0.004	Max. 0.015	balance

## Data Availability

The data presented in this study are available on request from the corresponding author. The data are not publicly available due to the nature of the company’s technological data.
